# Diagnostic pitfalls in functional neurological disorders

**DOI:** 10.1590/0004-282X-ANP-2022-S120

**Published:** 2022-08-12

**Authors:** Lucio Huebra Pimentel, Eduardo Genaro Mutarelli

**Affiliations:** 1 Universidade Federal de São Paulo, Departamento de Psicobiologia, São Paulo SP, Brazil;; 2 Universidade de São Paulo, Departamento de Neurologia, São Paulo SP, Brazil.

**Keywords:** Clinical Diagnosis, Somatoform Disorders, Diagnostic Errors, Diagnóstico Clínico, Transtorno Somatoformes, Erros de Diagnóstico

## Abstract

The diagnosis of functional neurological disorders is a major challenge in neurologist practice. Some clinical strategies can facilitate the recognition of functional disorders, but several pitfalls make their diagnosis difficult. Here we highlight the following points of attention during evaluation of patients with functional disorder: not all bizarre behavior is functional; not every event triggered by an emotional factor is a functional disorder; not every topographic incongruity is a functional disorder; patients may present functional and organic symptoms at the same time; psychiatric comorbid condition is not always evident in the history of a functional disorder; problematic communication at the time of diagnosis can compromise treatment and prognosis. In conclusion, we emphasize that special attention to these possible pitfalls facilitate the correct diagnosis and management of functional neurological disorders.

## RESUMO

O diagnóstico dos transtornos neurológicos funcionais é um grande desafio na prática do neurologista. Algumas estratégias clínicas podem facilitar o reconhecimento de transtornos funcionais, porém várias armadilhas dificultam o seu diagnóstico. Destacamos aqui os seguintes pontos de atenção durante a avaliação de pacientes com transtorno funcional: nem todo comportamento bizarro é funcional; nem todo evento desencadeado por um fator emocional é um transtorno funcional; nem toda incongruência topográfica é um transtorno funcional; pacientes podem apresentar ao mesmo tempo sintomas funcionais e orgânicos; condição psiquiátrica comórbida nem sempre é evidente na história de um transtorno funcional; comunicação problemática no momento do diagnóstico pode comprometer o tratamento e prognóstico. Em conclusão, enfatizamos que atenção especial a essas possíveis armadilhas, facilitam o diagnóstico e manejo corretos dos transtornos neurológicos funcionais.

## INTRODUCTION

 Studies of functional neurological disorders (FND) marked the beginning of modern neurology with its individualization in relation with psychiatry. FND is the second most frequent cause for neurological referrals after headache disorders^1^. In the emergency room setting, 9% of acute-onset neurologic symptoms are functional in origin^2^.

 Despite the high frequency of this condition, its psychopathological mechanisms are not fully understood and its diagnosis is difficult, with no confirmatory tests, generating discomfort and insecurity for the physician who is responsible for such evaluation.

 Functional disorders, especially due to their absence of organic damage and their psychic nature, carry great stigmas that can even affect the doctor-patient relationship, disturbing medical evaluation, and clinical judgment.

 In this review, we present some clinical pitfalls commonly seen in different scenarios of patient assessment with neurological functional disorders. We demonstrate a practical attitude, based above all on clinical experience.

## DEVELOPMENT

### Pitfall 1: Not every bizarre phenomenon is functional

 An almost always useful diagnostic tip in the evaluation of patients with suspected functional disorder is the search for inconsistencies in the neurological physical examination and the absence of a typical phenomenology, well recognized for an organic diagnosis. This strategy proves to be very effective, especially when evaluating motor symptoms, abnormal movements, gait alterations and paroxysmal events^3^. 

 A tremor with significant variation in frequency and amplitude, especially during distraction, raises the alarm for the functional nature of the disturbance. Just like the side-to-side head shaking during paroxysms events suggests psychogenic non-epileptic seizures. Gait with slow motion movements, important bending of the knees or sliding the feet as if skiing in the snow are bizarre movements, so different from the typical pattern of gait alterations due to organic diseases^4^. 

 The big pitfall we highlight in this case is that there are many exceptions for this rule. Not all bizarre phenomena, with difficult pathophysiological and topographical reasoning, are functional disorders.

 The first example to be mentioned is the alien hand syndrome. This motor symptom, classically found in cortico-basal degeneration, leads the patient to have sometimes complex, finalistic, but involuntary movements^5^. In these movements, the patient can pick up an object, touch another part of his body or even attack the examiner, without the slightest intention. This phenomenon could be confused with a functional disorder, especially when the involuntary motor act generates an unusual or embarrassing situation.

 Other conditions that may raise doubts about its organic nature are altered behaviors during sleep. Sleepwalking and nocturnal hypermotor epileptic seizures (originated more commonly from a focus in the frontal lobe) usually exhibit elaborate behaviors that may be difficult to distinguish from each other or even between these conditions and functional disorders^6^.

 Sometimes, functional disorders can be suspected in face of a disharmony between the level of concern and tension that the patient presents and the severity of his clinical condition, a fact classically described as “la belle indifference”^7^. But it is important to remember that in some situations the patient's affection may be altered, whether due to clinical diseases, medication effects or mental disorders^8^.

 Unmotivated laughter seems strange in situations of a medical consultation, but it can happen in a situation of some neurological diseases such as gelastic crises or pseudobulbar affection. Gelastic seizures are epileptic seizures with ictal laughter, most related to hypothalamic hamartoma, and more rarely related to cortical lesions^9^.

 Pseudobulbar affect is the neurological condition in which there is an inappropriate affective manifestation, disconnected with the mood and emotional state of that moment. The causes of pseudobulbar affection are structural lesions such as stroke, brain tumor, traumatic brain injury, inflammatory lesions such as multiple sclerosis and neurodegenerative diseases such as Parkinson's disease and Alzheimer's disease^10^.

### Pitfall 2: Not every event triggered by an emotional factor is a functional disorder

 Physicians tend to suspect functional disorders when symptoms are preceded by emotional triggers, especially negative emotions associated with a high stress load. Family problems, marital disputes, layoffs, and other psychosocial tensions are raised as precursors of functional disorders^11^. However, it is important to point out that there are organic conditions that can be precipitated or aggravated by stressful situations.

 One of the most commonly organic diseases related to emotional distress is neurally mediated syncope or vasovagal syncope. In addition to common triggers such as dehydration, heat exposure, sudden change in position, prolonged standing, and acute pain, emotional stressors also lead to on-off loss of consciousness^12^.

 Cataplexy is a cardinal symptom of narcolepsy type 1, integrating the classic symptomatic pentad. It is characterized by paroxysmal episodes of progressive loss of muscle tone triggered by emotional triggers, mainly positive stimuli, such as laughter or effusive happiness^13^.

### Pitfall 3: Not every topographical incongruity is a functional disorder

For a precise neurological diagnosis, it is important to define an accurate topographic diagnosis. In functional disorders, the signs found on the neurological examination are commonly not congruent to define a consistent or unique topographic location. However, it is important to note that some organic conditions can lead to multiple lesions, either in a monophasic or progressive evolution, leading to signs and symptoms that make a simpler topographic diagnosis difficult. Thus, it is important to think about a more complex topographic diagnosis, even multi-lesion disease before defining an incongruent topography of a functional diagnosis.

### Pitfall 4: Patients may have both functional and organic symptoms at the same time

 In addition to not being easy to define the diagnosis of a functional disorder, it may be not an exclusive diagnosis. It is possible and frequent the occurrence of functional and organic neurological disorders in the same patient simultaneously or at different times of life. Comorbid neurological conditions occur in approximately 20% of cases of functional disorders^14^.

 From the onset of the neurological symptoms, some patients may develop excessive preoccupation with their health status, including diagnosis and prognosis related to these symptoms, and develop new functional neurological symptoms or a worsening of that pre-existing symptom.

 It is also common for patients who present paroxysmal neurological symptoms such as epilepsy, syncope, cataplexy, parasomnias, and paroxysmal abnormal movements to present recurrent events of similar semiology.

### Pitfall 5: Psychiatric comorbid condition is not always evident in the history of a functional disorder

 During the evaluation of a patient with a functional disorder, it is common for the physician to look for the presence of a stressful event or the diagnosis of a mood disorder or psychiatric illness. About 2/3 to 3/4 of patients with FND have a psychiatric comorbidity, a rate much higher than other neurological diseases^15^.

 Not infrequently, it is difficult, especially in a single assessment, to point out an emotional trigger or define a psychiatric diagnosis. The absence of a more evident mental condition does not invalidate the diagnosis of a neurological functional disorder.

### Pitfall 6: Poor communication at the time of diagnosis can compromise treatment and prognosis

 The moment that generates the greatest difficulty for the physician in caring for a patient with a functional disorder is when communicating the nature of such a condition to the patients themselves and their family members. It is important to make clear the absence of organic substrate to justify the complaints, but without denying the existence of the symptoms and the suffering related to them^16^.

 A suggestion is always to present the patient with all the data that made the diagnosis possible, e.g. the Hoover’s sign, as well the possibility of reversing completely the symptoms due to the absence of structural damage^17^ ^,^ ^18^.

 One should never say that the patient does not have a disease. FND is now a “rule in” diagnosis. Try to show empathy with all the suffering of the patient and not to minimize that it is all related to some stress or anxiety, because at least a third of patients will deny any psychological problem.

 Failure in this communication generates difficulty in engaging in treatment, persistence of symptoms and seeking medical care with another professional, often restarting the cycle of intensive clinical evaluation and further investigation.

In conclusion, due to the hard diagnosis of FND, rational strategies must be developed to facilitate the best approach. We must always remember that neurological diseases may present with rare, bizarre, and sometimes difficult to understand phenomena, leading to a false suspicion of functional disorders. Emotional burden related to the event should not be the most valued point for diagnostic hypotheses. Once the diagnosis is defined, adequate communication guarantees a better clinical evolution. 
